# How Do Couple Relationship Interventions Improve Individual Well‐Being? The Role of Relationship Confidence

**DOI:** 10.1111/jmft.70104

**Published:** 2025-12-17

**Authors:** Noah B. Larsen, Allen W. Barton

**Affiliations:** ^1^ Department of Human Development & Family Studies University of Illinois Urbana‐Champaign Champaign Illinois USA

**Keywords:** couples, health, marriage, mechanism, program, relationship confidence, relationship education

## Abstract

Growing evidence suggests that couple relationship education (CRE) programs can improve not only relationship functioning but also individual health and well‐being. However, few studies to date have considered program factors that account for improvements in individual well‐being. The current study, in response, investigated three factors targeted by CRE programming (i.e., communication, partner support, relationship confidence) as potential mechanisms for changes in both individual and relationship outcomes. The sample comprised 340 individuals (170 couples) who participated in the online ePREP program with remote coaching and completed three waves of survey assessments spanning 6 months. Data analyses indicated that relationship confidence was significantly associated with improvements in multiple relationship and individual outcomes. No significant associations were observed between other program‐targeted factors (i.e., communication, partner support) and changes in individual outcomes. Findings offer implications for research and practice with couples, in particular, the wide‐reaching benefits of promoting individuals' relational efficacy.

## 1 Introduction

Couple and relationship education (CRE) programs continue to be widely disseminated throughout the United States. Recent reviews of high‐quality CRE programs generally support their effectiveness, noting benefits for areas of participants' relationship well‐being, including communication, relationship satisfaction, and relationship stability (Markman et al. 2022; Spencer and Anderson 2021). In recent years, a growing body of literature has also documented the positive effects of CRE programs for areas of individual health and well‐being, such as improved mental health, better sleep quality, and reduced substance use (Barton et al. 2021; Roddy et al. 2020; Spencer and Anderson 2021). The ability of CRE to produce benefits in these individual domains (despite no program content explicitly addressing such issues) is notable and highlights an additional public health benefit from such programs. Given these newly emerging findings, few studies to date have considered program factors that account for change in these individual domains among CRE participants. In response, the current study examined three potential mechanisms (i.e., communication, partner support, and relationship self‐efficacy) for improvements in both individual outcomes as well as relationship outcomes among a sample of CRE participants.

Although a significant body of research has investigated CRE program‐targeted mechanisms of effect, to date, much of this research has focused on mechanisms for relationship outcomes (e.g., satisfaction, stability), with significantly less attention devoted to mechanisms for individual outcomes. As a consequence, research is scant regarding the mechanisms by which participating in CRE programs may improve individual well‐being. In response, the present study examines three constructs targeted by CRE programs as potential mechanisms of effect. Among a sample of 170 couples (340 individuals) who participated in an online CRE program with remote coaching, researchers investigated whether these program‐targeted constructs forecasted 6‐month improvements in relationship and individual well‐being.

## 2 Literature Review

Given the prevalence of couple relationship distress and its adverse effect on adults, children, and communities (Grych and Fincham 1990; Kiecolt‐Glaser and Wilson 2017), CRE programs have been widely implemented and evaluated. Although varying in format and content, CRE programs primarily focus on helping couples and individuals develop behavioral, emotional, and cognitive skills related to positive relationship functioning (Stanley et al. 2020). As noted in the Introduction, prior meta‐analyses have documented that high‐quality CRE programs can confer benefits for areas of both couple relationship well‐being (Cohen's *d* = 0.10 for randomized controlled trial [RCT] designs, *d* = 0.47 for non‐RCT designs) as well as personal well‐being (e.g., mental health) (Cohen's *d* = 0.07 for RCT and non‐RCT designs) (Hawkins et al. 2022). In addition to investigating the effectiveness of CRE programs, prior research has also examined mechanisms of change in CRE programs (e.g., Beach et al. 2014; Stanley et al. 2007; Williamson et al. 2016). This body of literature highlights constructs such as relationship expectations, commitment, and communication as key purported mechanisms of CRE program effects (Stanley et al. 2020). Various studies highlight support for the effects of these mechanisms (e.g., Barton et al. 2017; Beach et al. 2014; Bodenmann et al. 2009; Roddy et al. 2020), though some studies also report mixed, or null, findings for these mechanisms (e.g., Owen et al. 2013; Stanley et al. 2007; Williamson et al. 2016). Given these ongoing mixed results, relationship scholars continue to emphasize the need for additional scientific investigation of mechanisms of effect in CRE programs (Markman et al. 2022; Stanley et al. 2020; Wadsworth and Markman 2012).

With the expanding evidence of CRE program effects for individual health and well‐being, one particular call for future research in this area has been for a better understanding of mechanisms associated with improvements in individual outcomes, specifically (Barton et al. 2021; Roddy and Doss 2020). While it is increasingly clear *that* certain CRE programs can promote individual well‐being, it still remains rather unclear *why* CRE programs have these effects. In addition to answering a scientific area of inquiry, practically, identifying program‐related constructs associated with individual well‐being offers key insights for program developers and evaluators regarding which components are most salient to CRE programs that seek to also promote individual well‐being (Pearson et al. 2005; Van Epp 2016). To this end, the present study examines three constructs commonly targeted by CRE programs—communication, partner support, and relationship confidence—as mechanisms for change for individual and relationship outcomes. A brief review of each of these potential mechanisms follows.

### 2.1 CRE Mechanisms of Effect for Couple Well‐Being Outcomes

#### 2.1.1 Communication

Communication has traditionally been a main focus of CRE programs. Attention to communication in CRE programs draws upon behavioral theories of relationship change (Markman and Floyd 1980; Weiss 1984) and empirical findings that patterns of communication can enhance or weaken relationships (e.g., Gottman and Notarius 2000; Markman 1981). The premise of many present‐day CRE programs is that couples' communication is a mechanism to create more stable and satisfactory relationships (Stanley et al. 2020). However, recent empirical studies examining communication as a mechanism of relationship change in CRE programs have produced inconsistent results (e.g., Bradbury and Bodenmann 2020; Wood et al. 2014). Although some studies confirm (e.g., Barton et al. 2017; Beach et al. 2014; Roddy et al. 2020), others provide limited evidence (Stanley et al. 2007; Williamson et al. 2016) or even contradict (Baucom et al. 2006; Schilling et al. 2003) the notion that program improvements in couple communication are reliably associated with improvements in relationship well‐being. Thus, while communication remains a primary targeted outcome for CRE programs, and an area where couples report the most benefit from participating in CRE programs (Burr et al. 2014; Stanley et al. 2001), the degree to which improved communication is linked to other areas of relationship change has mixed findings.

#### 2.1.2 Partner Support

The scope of CRE programs is not limited to communication, however. Many CRE programs also emphasize emotional support between partners as a mechanism of change, as perceptions of support can contribute to the developmental course of relationships generally (Bradbury et al. 2000). In prior research with couple interventions, improvements in felt acceptance and intimate safety (Hawrilenko et al. 2016) as well as emotional intimacy (Roddy et al. 2020) were associated with concurrent improvements in relationship satisfaction. Furthermore, program improvements in individuals' emotional support have also been found to be associated with the maintenance of program reductions in break‐up potential (but not relationship satisfaction) (Le et al. 2020).

#### 2.1.3 Relationship Confidence

Individuals' self‐efficacy and confidence in handling relationship challenges represents another common construct targeted by CRE programs. Attention to this construct stems from tenets of social cognitive theory (Bandura and McClelland 1977), a theoretical framework that informs many CRE programs. Social cognitive theory highlights the importance of cognitive processes in behavior change, emphasizing that individuals are more likely to engage in behaviors when they hold the belief that they can perform them successfully and achieve a positive outcome. To that end, social cognitive theory emphasizes the importance of CRE programs instilling in participants a sense of efficacy and confidence about their relationship, ability to manage relationship problems, and have a successful relationship (Stanley 2013). Multiple studies have documented that couples often gain more confidence in their relationship during the course of CRE programs (for reviews, see Markman et al. 2022; Markman and Rhoades 2012; Spencer and Anderson 2021), yet little research has tested relationship confidence as a program‐targeted mechanism of future relationship well‐being. As an exception, a recent study indicated that program improvements in relationship confidence were associated with the maintenance of pre‐post improvements in break‐up potential. Relationship confidence, however, was not associated with maintenance of improvements in relationship satisfaction, a pattern similar to findings on emotional support as a mechanism of effect (Le et al. 2020).

### 2.2 CRE Mechanisms of Effect for Individual Well‐Being Outcomes

As noted in the Introduction, multiple recent studies highlight the ability of CRE programs to also confer benefits for areas of individual well‐being in addition to relational well‐being. Meta‐analytic findings suggest that effect sizes are generally smaller for individual outcomes versus relationship outcomes (Hawkins et al. 2022), although sizeable benefits have been noted for participants' psychological distress (*d* = −0.36), sleep problems (*d* = −0.24), and problematic alcohol use (*d* = −0.11) in prior RCTs for CRE programming (Roddy et al. 2020). To date, however, research remains limited regarding the mechanisms by which participating in such programs may improve individual well‐being outcomes. This gap persists despite scholars noting that the advancement of the CRE field depends not only on testing program efficacy but also on identifying which program factors contribute to positive outcomes (Stanley et al. 2020; Wadsworth and Markman 2012).

Studies by Roddy et al. (2020), Barton et al. (2021), and Cooper et al. (2021) offer three of the most direct studies to date on mechanisms by which participating in CRE programs may improve individual well‐being outcomes. Roddy et al. (2020) found that CRE program participation indirectly improved perceived health through increased emotional support as well as, separately, through reduced communication conflict. In addition to these indirect effects, study findings also indicated direct, concurrent associations between improvements in program‐targeted constructs (i.e., communication conflict, emotional support) and improvements in other areas of individual well‐being (i.e., psychological distress, alcohol use, and insomnia). Barton et al. (2021) examined an aggregate measure of couple functioning—composed of communication, partner support, relationship confidence, and couple satisfaction—and found support for its ability to link CRE program assignment to changes in individuals' mental health, sleep, and overall general health. Similarly, Cooper et al. (2021) used a composite measure of couple functioning and observed that post‐program couple functioning predicted subsequent gains in general mental health.

Building on these findings, the present study sought to more clearly understand specific constructs that contribute to CRE effects on individual well‐being. Although limited, prior theoretical and empirical writings suggest that the three aforementioned mechanisms for couple outcomes—communication, partner support, and relationship confidence—may also function as program‐targeted factors that meaningfully affect individual outcomes. The Strengths and Strains Model of Marital Quality and Physical Health (Slatcher 2010), for instance, theoretically highlights how positive (e.g., support, positive communication) and negative (e.g., conflict) areas of relationship functioning exert direct effects on individuals' psychological well‐being and health behaviors (e.g., sleep, substance abuse). Empirical research studies on couple relationship functioning highlight the direct effects of couple communication for individuals’ depressive symptoms (Beach et al. 1998; Fincham and Beach 1999), problematic alcohol use (Murphy and O'Farrell 1994), and sleep disturbances (Troxel et al. 2007). Similarly, partner support has documented associations with indicators of mental health (Vaingankar et al. 2020) and physiological stress recovery (Heffner et al. 2004). Lastly, relationship confidence has also been linked to individual outcomes such as depressive symptoms (Whitton et al. 2007) and general health (Barton et al. 2018). Taken together, these findings support the idea that such relational factors would affect individual outcomes in a CRE context as well, which the present study aimed to address.

Building on these findings, the current study sought to address the following research question: Do communication, partner support, and/or relationship confidence function as mechanisms contributing to improvements in both individual and relationship outcomes among CRE participants? Informed by research and theory showing the importance of specific areas of relationship functioning for the well‐being of individuals and couples (Bradbury et al. 2000; Markman 1981; Robles et al. 2014; Slatcher 2010), we examined the hypotheses that greater levels of (a) communication, (b) partner support, and (c) relationship confidence following CRE programming would be associated with improvements in relationship outcomes (H1a–c) and individual outcomes (H2a–c). Study models were run with all three targeted mechanisms in order to identify the specific, unique effects of each construct, controlling for the presence of others.

## 3 Method

### 3.1 Participants

Program interest forms were completed by 618 individuals, and consent forms and baseline surveys were completed by 281 of these respondents and 220 partners. From this, 215 couples (430 individuals) had consent forms completed by both partners, and 170 couples (340 individuals) attended the first scheduled coach call and were officially enrolled in the program. From this sample of 340 respondents, the average age was 39.53 (SD = 12.19). Approximately two‐thirds of the sample (66%) were married, 24% were dating and living together, 9% were engaged, and 1% were married and currently separated. Eighty‐one percent of the participants identified as White, 9% as Black or African American, 5% as Asian, 1% as American Indian or Alaska Native, 1% as Native Hawaiian or other Pacific Islander, and 5% as other. The median family income was in the range of $85,000 to $95,000, and the median number of children was two.

Wave 2 surveys were completed by 284 individuals (84% retention), including 153 couples (90% retention) with at least one respondent. Wave 3 surveys were completed by 257 individuals (76% retention), with 141 couples (83% retention) including at least one respondent.

### 3.2 Procedure

Individuals could learn about the project through various means, including online advertisements, Extension communications, media features, and word of mouth. Data collection spanned from October 2020 to June 2023. All promotional material directed individuals to the project website, which provided individuals with information about the project and a brief program interest form. Upon receipt of a program interest form, individuals were sent an email that contained a link to the consent form and baseline survey as well as a separate email to forward to their partner with this information. To be eligible, individuals had to be 18 years of age or older; married, engaged, or in a cohabiting relationship for at least 6 months; resident of Illinois; willing to participate in the relationship education program; and in a relationship in which neither partner reported the occurrence of severe intimate partner violence.

Upon receipt of both partners' consent forms and baseline surveys, couples were assigned a program coach who then contacted the couple about scheduling their first coach call. Once the first coach call had occurred and eligibility was confirmed, the couple was enrolled, sent a link to access the ePREP program, and mailed program summary material. Couples were encouraged to complete one session per week, with additional coach calls scheduled to occur after completion of every two sessions for a total of four coach calls. A booster (fifth) coach call was planned for 1 month following program completion (see Program Content and Delivery section for additional programmatic details).

The Wave 2 survey was emailed to participants within a few days of program completion (i.e., after coach call four). Couples that did not finish the program were sent the Wave 2 survey at a similar time interval (i.e., approximately 2 months following enrollment). The Wave 3 survey was emailed approximately 6 months after enrollment. Each survey took about 15 min to complete. Eligible individuals were paid $20, $25, and $30 for completing the first, second, and third surveys, respectively. Individuals were not compensated for program attendance or completion. The study was approved by the Institutional Review Board of the sponsoring research university (Protocol #20837; Title: Illinois United: ePREP program evaluation).

### 3.3 Program Content and Delivery

The Illinois Strong Couples project involved a dissemination of the ePREP program, a universal (cf. selective or indicated) prevention program that all couples can participate in irrespective of level of distress. The efficacy of this program is well‐established (Braithwaite and Fincham 2009, 2011), including in a recent ACF‐funded trial involving ePREP with remote coaching provided by licensed clinicians or graduate students in clinical psychology or marriage and family therapy (Doss et al. 2020). The ePREP program was selected for the current dissemination project given its universal prevention program design, well‐documented efficacy, and appearance in prior studies examining individual outcomes following couple relationship education (Braithwaite and Fincham 2007, 2011, 2014; Roddy et al. 2020). The online ePREP version (vs*.* in‐person PREP program) was chosen as a way to reduce logistical, geographic, and financial constraints associated with in‐person programming and in light of findings that individuals often seek online resources when hoping to improve their relationship (Barton and Larsen, in press; Trillingsgaard et al. 2019). The ePREP program consists of six online sessions, each approximately 45–60 min in length. Each session included psychoeducational presentations, videos featuring example couples, and discussion questions. Program content covers various topics including communication, conflict, problem‐solving, commitment, and friendship. Recommended homework assignments were emailed to couples following each session. Couples were strongly encouraged to watch the online content together.

Program coaching followed a structured manual originally created by the developers of ePREP and utilized in prior research (Doss et al. 2020). Coach calls did not introduce any new material or concepts, but rather served to ensure proper understanding of program content, provide opportunities to practice and apply program content to their relationship, and encourage couples in their efforts to strengthen the relationship. As one adaptation from prior implementation, a booster (i.e., fifth) coach call was developed and offered to participants approximately 1 month after the completion of the ePREP sessions and 4th coach call. This fifth call was designed to reinforce material covered in the program and encourage couples in their continued application of the material.

Extension educators and graduate students in Clinical/Community Psychology from the land‐grant institution of the implementing state were trained as coaches for the program. A total of 10 Extension Educators and 7 graduate students from the sponsoring university participated as coaches. At the beginning of the project (prior to training additional coaches), the project director and two coaches from a prior dissemination of this approach assisted with coaching when enrollment exceeded existing coaching capacity. No Extension educators or Psychology graduate students had any prior exposure to the ePREP program or training in CRE programming and program delivery. All coaches participated in an eight‐session training program conducted by the project director prior to working with participating couples. Most coaches also conducted coach calls with a practice couple before beginning formal coaching. Coaches participated in weekly or bi‐weekly coach supervision meetings. Calls were recorded, and a random sample of coach calls was scored for fidelity.

Prior analyses of this project found significant improvements over time in multiple domains of relationship functioning (i.e., communication conflict, partner support, relationship instability, and coparenting conflict) as well as individual functioning (i.e., psychological distress, sleep problems, problematic alcohol use, and general health perceptions) (Barton et al. 2024). Relationship confidence and couple satisfaction were not examined as constructs in this study. Additionally, published procedural findings have highlighted factors contributing to program enrollment and attendance as well as participants’ high levels of satisfaction with the program (Barton and Larsen in press).

### 3.4 Measures

#### 3.4.1 Romantic Relationship Outcomes

##### 3.4.1.1 Couple Satisfaction

Individuals’ relationship satisfaction was assessed at Waves 1, 2, and 3 using the four‐item version of the Couple Satisfaction Index (Funk and Rogge 2007). An example item includes, “How rewarding is your relationship with your partner?” Response options ranged from 0 = *Extremely unhappy* to 6 = *Perfect* (question 1), 0 = *Not at all true* to 5 = *Completely true* (question 2), and 0 = *Not at all* to 5 = *Completely* (questions 3–4). Items were summed, with higher scores representing higher relationship satisfaction (*α* ≥ 0.93).

##### 3.4.1.2 Relationship Instability

Potential dissolution of the relationship was assessed at Waves 1, 2, and 3 using three items adapted from the Marital Instability Index (Edwards et al. 1987). Individuals were asked to report the frequency of past month thinking about the following three items: “The thought of ending my relationship has crossed my mind,” “I've thought my relationship might be in trouble,” and “How likely is it that you and your partner will break up within the next year?” Response options ranged from 1 = *Never in the past month* to 5 = *More than once a day* (questions 1 and 2) and 1 = *Very unlikely* to 5 = *Very likely* (question 3). Items were summed such that higher scores indicate greater relationship instability (*α* ≥ 0.87).

#### 3.4.2 Individual Outcomes

##### 3.4.2.1 Psychological Distress

Psychological distress was assessed at Waves 1, 2, and 3 using six items from the Kessler Psychological Distress Scale (Kessler et al. 2002). A sample item from this scale asks respondents how frequently in the past 30 days they had felt that everything was an effort. Response options ranged from 1 = *None of the time* to 5 = *All of the time*. Internal consistency in the current sample was good (*α* ≥ 0.89).

##### 3.4.2.2 Sleep Problems

Quality of sleep was assessed by seven items using the Insomnia Severity Index at Waves 1, 2, and 3 (Bastien 2001). A sample item is, “How satisfied/dissatisfied are you with your current sleep pattern?” (0 = *Very satisfied* to 4 = *Very dissatisfied*). All items were summed, with higher scores indicating greater sleep difficulties (*α* ≥ 0.88).

##### 3.4.2.3 Problematic Alcohol Use

The seven‐item PROMIS Alcohol Use measure was used to assess problematic alcohol use in the past month (Pilkonis et al. 2013). Participants reported at Waves 1, 2, and 3 on a five‐point Likert scale about their alcohol use behaviors (1 = *Never* to 5 = *Almost always*). A sample item is, “In the past 30 days, I was unreliable after I drank.” Items were summed with higher numbers indicative of greater problems (*α* ≥ 0.88).

#### 3.4.3 Program‐Targeted Constructs

##### 3.4.3.1 Communication Conflict

Experience of negative communication was assessed at Waves 1, 2, 3 using seven items created for the Administration for Children and Families (ACF) Healthy Marriage Initiative (Doss et al. 2020). Participants were asked to rate how frequently communication conflict occurred during the past month on a Likert scale. Response options ranged from *Never* (1) to *Often* (4). A sample item includes, “My partner/spouse blamed me for his/her problems.” All items were summed, with higher scores indicating greater communication conflict. Cronbach's *α* was 0.92.

##### 3.4.3.2 Partner Support

Partner support was measured at Waves 1, 2, 3 by five items developed for the ACF (Doss et al. 2020). Participants reported how much support they received from their partner on a four‐point Likert scale (1 = *Strongly disagree* to 4 = *Strongly agree*). Example items include “I feel appreciated by my partner/spouse” and “My partner/spouse expresses love and affection toward me.” All the items were summed; higher scores on this scale represent higher perceived partner support (*α* = 0.86).

##### 3.4.3.3 Relationship Confidence

Individuals' confidence in the future of their relationship was measured using four items from the Relationship Confidence Scale (Stanley et al. 1994). At Waves 1, 2, 3, each item was measured on a Likert scale ranging from 1 = *Strongly disagree* to 5 = *Strongly agree*. Sample items included, “I feel good about my and my partner's prospects to make this relationship work for a lifetime” and “My partner and I have the skills a couple needs to make a relationship last”. Cronbach's *α* was 0.93.

### 3.5 Plan of Analysis

Study analyses were conducted using a series of regression models. Given the lack of any gender‐specific hypotheses, analyses occurred at the individual level, with individuals nested within dyads using the “cluster” command in Mplus Version 8.3 (Muthén and Muthén 2017). Regression models were conducted to examine associations between program‐targeted constructs at program conclusion and 6‐month changes in couple well‐being and individual well‐being. For relationship outcomes, two regression models were run, corresponding to each couple well‐being outcome (i.e., couple satisfaction, relationship instability). For individual outcomes, three regression models were run, which corresponded to the three individual health outcomes under investigation (i.e., psychological distress, sleep issues, problematic alcohol use). We entered all program‐targeted constructs (i.e., communication conflict, partner support, relationship confidence) as predictor variables in each model to assess the unique predictive ability of each construct.

All regression models also controlled for various demographic factors (i.e., participant sex, age, education level, household income, and marital status), given evidence of their influence on relationship education outcomes (e.g., see Stanley et al. 2020). Missing data for all study variables were handled using full information maximum likelihood estimation in Mplus 8.3 (Muthén and Muthén 2017). Little's MCAR test indicated that data were not missing completely at random (chi‐square = 301.40, *df* = 246, *p* < 0.01). Follow‐up analyses indicated that respondents at subsequent waves were more likely to be married as well as report a higher income and level of education compared to nonrespondents. Respondents also reported higher levels of couple satisfaction as well as lower levels of relationship instability and psychological distress at Wave 1. All aforementioned variables were already planned to be included as control variables in analyses.

## 4 Results

### 4.1 Descriptive Statistics

Table 1 presents descriptive statistics and correlations for study variables. Correlations were in the expected directions. Using conventional cut‐off criteria for relationship distress according to the Couple Satisfaction Index (< 13.5; Funk and Rogge 2007) 63% of individuals were relationally distressed at Wave 1. Mean scores of individual well‐being indicated generally low to moderate levels of psychological distress and sleep problems, with the mean score for problematic alcohol use at a relatively low level.

**Table 1:** Correlation matrix and descriptive statistics for study variables (*N* = 340).

Variable	1	2	3	4	5	6	7	8	9	10	11	12	13	14	15	16	17	18
1. Couple satisfaction (W1)	−																	
2. Relationship instability (W1)	−0.69**	−																
3. Psychological distress (W1)	−0.49**	0.55**	−															
4. Sleep problems (W1)	−0.26**	0.27**	0.49**	−														
5. Problematic alcohol use (W1)	−0.19**	0.24**	0.20**	0.17**	−													
6. Communication conflict (W2)	−0.51**	0.42**	0.33**	0.17**	0.12	−												
7. Partner support (W2)	0.62**	−0.51**	−0.35**	−0.15*	−0.13	−0.56**	−											
8. Relationship confidence (W2)	0.60**	−0.54**	−0.35**	−0.15*	−0.14*	−0.61**	0.78**	−										
9. Couple satisfaction (W3)	0.73**	−0.49**	−0.41**	−0.31**	−0.15*	−0.56**	0.69**	0.68**	−									
10. Relationship instability (W3)	−0.50**	0.52**	0.40**	0.22**	0.18*	0.45**	−0.51**	−0.58**	−0.71**	−								
11. Psychological distress (W3)	−0.37**	0.35**	0.71**	0.44**	0.25**	0.28**	−0.29**	−0.36**	−0.48**	0.53**	−							
12. Sleep problems (W3)	−0.21**	0.14*	0.41**	0.74**	0.06	0.15*	−0.15*	−0.21**	−0.29**	0.25**	0.52**	−						
13. Problematic alcohol use (W3)	−0.09	0.12	0.19**	0.17*	0.63**	0.14	−0.10	−0.17*	−0.18*	0.22**	0.33**	0.21**	−					
14. Sex (1 = female)^a^	0.03	0.16**	0.11	0.06	−0.05	−0.05	−0.08	−0.04	0.02	−0.01	−0.07	0.00	−0.12	−				
15. Age	−0.05	−0.11*	−0.22**	0.05	−0.06	0.06	−0.06	−0.03	−0.12	−0.10	−0.14*	0.08	−0.03	−0.07	−			
16. Education level	0.18**	−0.22**	−0.17**	−0.10	−0.13*	−0.12*	0.17**	0.09	0.17*	−0.19**	−0.27**	−0.15*	−0.02	0.15**	0.08	−		
17. Income	0.18**	−0.29**	−0.30**	−0.16**	−0.09	−0.14*	0.22**	0.16**	0.18**	−0.22**	−0.35**	−0.16*	−0.01	−0.03	0.22**	0.43**	−	
18. Marital status (1 = married)^a^	−0.05	−0.19**	−0.13*	−0.02	−0.16*	−0.04	−0.02	0.14*	−0.06	−0.18**	−0.09	0.03	−0.16*	0.01	0.33**	0.13*	0.18**	−
Mean	11.98	5.95	13.61	12.57	9.24	15.38	16.15	16.21	14.04	4.37	11.34	11.07	8.18	0.51	39.53	8.23	5.06	0.66
SD	4.62	2.91	5.37	5.70	4.27	5.37	3.01	3.32	4.38	2.16	4.92	5.44	2.60	0.50	12.19	1.91	2.98	0.47
Minimum	0	3	6	4	7	7	5	8	0	3	6	4	7	0	21	2	0	0
Maximum	21	15	30	29	35	28	20	20	21	15	30	29	24	1	80	11	8	1
% missing	2	0	0	0	24	20	20	20	28	28	27	27	47	0	0	0	1	< 1

^a^ Spearman correlation.

** *p* < 0.01

* *p* < 0.05.

### 4.2 Associations Between Program‐Targeted Constructs and Relationship Outcomes

The first set of analyses examined associations between program‐targeted constructs (i.e., relationship confidence, partner support, communication conflict) at post‐program and 6‐month changes in relationship well‐being outcomes using multiple regression models. These results are summarized in Table 2. Two models were tested, corresponding to the two couple relationship outcomes examined (i.e., couple satisfaction, relationship instability). Results are organized by each predictor variable. Analyses indicated that communication conflict was not significantly associated with couple satisfaction (*B* = −0.04, *p* = 0.46) or relationship instability (*B* = 0.03, *p* = 0.32). Heightened partner support was associated with significant increases in couple satisfaction (*B* = 0.34, *p* < 0.01) but was not associated with significant changes in instability concerns (*B* = −0.09, *p* = 0.26). Heightened relationship confidence was associated with significant declines in relationship instability concerns (*B* = −0.17, *p* < 0.05), and the association between greater relationship confidence and increases in couple satisfaction was at the threshold for statistical significance (*B* = 0.25, *p* = 0.05). As shown in Table 2, demographic control variables (i.e., sex, age, education level, income level, and marital status) were not significantly associated with changes in any relationship outcomes. In sum, results provided support for Hypothesis 1c and partial support for Hypothesis 1b, indicating relationship confidence and, to a lesser extent, partner support as factors contributing to changes in relationship outcomes.

**Table 2:** Regression models for associations between program‐targeted constructs and well‐being outcomes.

	Relationship outcomes (W3)		Individual outcomes (W3)		
	Couple satisfaction^a^	Relationship instability^a^	Psychological distress^b^	Sleep problems^b^	Problematic alcohol use^c^
Program‐targeted construct	*B* (*β*) (*SE*)	*B* (*β*) (*SE*)	*B* (*β*) (*SE*)	*B* (*β*) (*SE*)	*B* (*β*) (*SE*)
Communication conflict (W2)	−0.04 (−0.05) (0.05)	0.03 (0.07) (0.03)	0.00 (0.00) (0.06)	−0.09 (−0.09) (0.07)	−0.01 (−0.02) (0.04)
Partner support (W2)	0.34 (0.24)** (0.12)	−0.09 (−0.14) (0.08)	0.13 (0.08) (0.15)	0.08 (0.04) (0.17)	0.07 (0.07) (0.10)
Relationship confidence (W2)	0.25 (0.19)+ (0.13)	−0.17 (−0.27)* (0.07)	−0.26 (−0.17)* (0.12)	−0.29 (−0.17)* (0.14)	−0.15 (−0.18) (0.11)
Control variables					
Sex (1 = female)	−0.11 (−0.01) (0.31)	−0.06 (−0.02) (0.16)	−0.66 (−0.07)+ (0.38)	−0.20 (−0.02) (0.48)	−0.23 (−0.04) (0.27)
Age	−0.01 (−0.02) (0.02)	−0.02 (−0.09)+ (0.01)	0.01 (0.01) (0.02)	0.01 (0.02) (0.02)	0.00 (−0.01) (0.02)
Education level	−0.02 (−0.01) (0.12)	0.00 (0.00) (0.05)	−0.35 (−0.13)* (0.15)	−0.15 (−0.05) (0.15)	0.01 (0.01) (0.10)
Income	0.01 (0.00) (0.09)	0.02 (0.03) (0.05)	−0.14 (−0.08) (0.11)	−0.09 (−0.05) (0.10)	0.11 (0.10) (0.07)
Marital status (1 = married)	0.01 (0.00) (0.57)	−0.50 (−0.11) (0.36)	−0.21 (−0.02) (0.58)	0.40 (0.03) (0.59)	−0.35 (−0.06) (0.51)
Baseline (W1 of dependent variable)	0.41 (0.43)** (0.08)	0.22 (0.28)** (0.06)	0.61 (0.63)** (0.06)	0.68 (0.71)** (0.05)	0.40 (0.61)** (0.12)
*R* ^2^	0.62**	0.44**	0.54**	0.55**	0.42**

*Note:* W1 = Wave 1; W2 = Wave 2; W3 = Wave 3.

^a^ *N* = 229.

^b^ *N* = 231.

^c^ *N* = 157.

* *p* < 0.05.

** *p* < 0.01.

+ *p* < 0.10.

### 4.3 Associations Between Program‐Targeted Constructs and Individual Outcomes

Having examined associations between program‐targeted constructs and changes in relationship well‐being, the next set of analyses tested associations with individual well‐being. Results from these analyses are summarized in Table 2. A total of three models were tested, corresponding to each of the three individual outcome variables (i.e., psychological distress, sleep problems, and problematic alcohol use). Similar to the first set of analyses, results are organized with respect to each predictor variable. Communication conflict was not associated with any significant changes in psychological distress (*B* = 0.00, *p* = 0.10), sleep problems (*B* = −0.09, *p* = 0.16), or problematic alcohol use (*B* = −0.01, *p* = 0.79). Similarly, partner support was not linked to any significant changes in psychological distress (*B* = 0.13, *p* = 0.38), sleep problems (*B* = 0.08, *p* = 0.65), or problematic alcohol use (*B* = 0.07, *p* = 0.48). Heightened relationship confidence was associated with significant declines in psychological distress (*B* = −0.26, *p* < 0.05) and sleep problems (*B* = −0.29, *p* < 0.05), but was not associated with changes in problematic alcohol use (*B* = −0.15, *p* = 0.15). As shown in Table 2, among demographic control variables, a significant association appeared only with respect to education level. Possessing a higher level of education was associated with greater declines in psychological distress (*B* = −0.35, *p* < 0.05). Demographic variables were not significantly associated with any changes in sleep problems or problematic alcohol use. In summary, for individual well‐being, relationship confidence was associated with significant improvement in two outcomes, providing support for Hypothesis 2c. No significant associations were observed between other program‐targeted constructs (i.e., communication conflict or partner support) and any individual outcome. Thus, results did not support Hypotheses 2a or 2b.

### 4.4 Post‐Hoc Analyses

Given the significant effects observed with relationship confidence as well as the limited prior empirical investigations of this construct in CRE programming, additional post hoc analyses were conducted with this construct. These analyses investigated: (a) whether program participants demonstrated significant increases in relationship confidence following program participation, as well as (b) sociodemographic predictors of change in relationship confidence to ascertain whether particular individuals or relationship types were more likely to report improvements in relationship confidence. Results of these post‐hoc analyses indicated: (a) participants reported significant increases in relationship confidence over the course of the program of medium effect size (*d* = 0.63), and (b) levels of improvement in relationship confidence were similarly reported across participant income, age, education level, and sex; differences were only observed for marital status, with married individuals reporting greater improvements in relationship confidence at the end of the program compared to nonmarried individuals. For additional details and tabulated findings for post‐hoc analyses, please refer to Supporting Information S1: File S1.

## 5 Discussion

Findings from the present study add to the growing literature on the benefits of CRE programs by documenting program‐targeted constructs that predicted improvements in relationship and individual well‐being, the latter being a heretofore relatively understudied area. Notably, multiple program‐targeted mechanisms were examined simultaneously, and effects were tested for multiple indicators of relationship and individual well‐being. Collectively, findings expand prior theoretical and empirical work on factors that account for improvements following CRE participation, particularly with respect to benefits for individual well‐being. Across all examined models, relationship confidence (vs. communication conflict or partner support) demonstrated the greatest number of significant associations with postprogram improvements. Results suggest that developing individuals' efficacy and confidence in achieving relationship success may be an important area of consideration for CRE programs seeking to improve areas of personal well‐being in addition to relationship well‐being.

The importance of relationship confidence for predicting changes in long‐term study outcomes is consistent with central tenets of Bandura's (1977) social cognitive theory, a framework that has informed many preventive efforts for couples and families as a whole. Although the utility of this framework for couple interventions has been questioned by some (e.g., Johnson and Bradbury 2015), current results lend support to a process whereby CRE programs that foster individuals' efficacy in handling relationship challenges and achieving relationship success can, in turn, foster subsequent positive change in various domains. That confidence about one's relationship beneficially spills over and shapes individuals' emotional and physical states over time also aligns with basic research on couple relationships (e.g., Whitton et al. 2007). Prior basic research studies note clear associations between heightened levels of destructive relationship possesses and worsened mental health, sleep, and substance use (Derrick et al. 2019; Kiecolt‐Glaser and Wilson 2017; Robles et al. 2014); as such, the corollary may also prove true, such that confidence and optimism for one's relationship is associated with greater personal well‐being, at least among this sample of help‐seeking couples.

By testing multiple program‐targeted constructs (cf. an aggregated or global measure of relationship quality) (see Barton et al. 2021; Cooper et al. 2021), these results provide greater understanding of how certain aspects of CRE programs influence well‐being. Indeed, there were notable differences regarding which program‐targeted constructs were associated with improvements in couple well‐being (see also Le et al. 2020; Roddy et al. 2020). That partner support was positively associated with improvements in couple satisfaction extends findings from prior research documenting concurrent associations between changes in emotional support and couple satisfaction (Hawrilenko et al. 2016; Roddy et al. 2020), suggesting improvements in this more positively‐laden aspect of couple functioning may be particularly relevant for shaping individuals’ overall relationship satisfaction.

With respect to other program‐targeted mechanisms, prior research found that program improvements in communication conflict and partner support were associated with concurrent improvements in areas of individual well‐being (Roddy and Doss 2020). However, in the current study, neither postprogram partner support nor communication conflict was significantly associated with changes in any of the individual outcomes, despite the use of similar measures as the aforementioned study. Perhaps unlike day‐to‐day relationship behaviors (e.g., support, communication), relationship confidence may encompass a broader sense of security and future in the relationship that provides unique benefits over time for one's mental health, sleep, and substance use. Communication conflict was not significantly associated with any outcome, which is consistent with studies that found a null effect for observed communication as a mechanism of program effect on relationship quality (Stanley et al. 2007; Williamson et al. 2016). However, given significant bivariate correlations between communication and the majority of study outcomes, it is possible that, although important, the effect of communication on changes in these outcomes is diminished once post‐program levels of relationship confidence and partner support are accounted for.

Post‐hoc tests investigated the degree of improvement in relationship confidence following program participation, and whether certain groups experienced more or less program improvement. Results demonstrate clear increases in participants’ relationship confidence over the course of the program. Few sociodemographic factors forecasted program improvements in relationship confidence, suggesting that gains in this area were likely to appear across a variety of individuals and relationship types. The one exception was marital status, with married individuals reporting greater increases. This finding is consistent with prior research that has found married couples to report greater improvements relative to unmarried participants in CRE programming (Barton et al. 2023). In this way, married couples—those who have already made a public, formal commitment to the longevity of the relationship—may benefit more from certain aspects of CRE programming, as they are engaging in efforts to strengthen this relationship that is consistent with this previous (and perhaps current) conviction (see also Stanley et al. 2010).

### 5.1 Limitations

Results from the present study should be considered in light of certain limitations. First, the study design did not include a control group. As a result, mechanistic effects cannot be empirically documented as exclusively due to program participation in the absence of a control condition. Future research using an experimental design can test whether similar mechanisms of interest mediate the effects of program participation on individual outcomes. Second, data collection spanned only 6 months. Collection of longer‐term follow‐up assessments of well‐being outcomes would allow for testing whether associations between mechanisms of interest and outcomes were enduring. Third, the study required participation from both partners in each dyad, potentially resulting in a sample with greater levels of relationship functioning compared to the general population (Barton, Hatch, et al. 2020; Barton, Lavner, et al. 2020). Fourth, all measures were based on self‐report. Future research including observations of couple functioning and more extensive assessments of personal health, including biomarkers, would be valuable. Fifth, given generally low levels of alcohol misuse among the current sample (cf. clinical or treatment samples), limited variability in this construct relative to the other outcomes may have precluded the ability to detect significant associations with program‐targeted mechanisms. Finally, as with research on any CRE program, findings from the current study may be specific to the ePREP program and may not be generalizable to all CRE programs.

### 5.2 Conclusion

Notwithstanding these limitations, the present study provides novel contributions to scholarship on CRE programming. Study findings highlight specific areas of relationship functioning that can be targeted by researchers and practitioners in future work with CRE programs as well as in basic research seeking to understand how particular couple relationship processes shape personal health. Findings from the present study highlight the salience of relationship confidence as a CRE program mechanism of effect for relationship and individual well‐being outcomes, warranting further research on this topic. In addition, practitioners and program administrators may consider devoting particular attention to fostering relationship confidence among partnered individuals. Given the widespread implementation and evaluation of CRE programs and growing evidence of their collateral benefits for individual health, testing mechanisms of effect for individual health outcomes remains a key area for research.

## Supporting information

Supplemental‐File‐Clean‐Unlinked.

## Data Availability

Data and analytic syntax are available upon request from the first author.
