# Supplementary material for: How Do Couple Relationship Interventions Improve Individual Well‐Being? The Role of Relationship Confidence
Source: J Marital Fam Ther. 2025 Dec 17;52(1):e70104. doi: 10.1111/jmft.70104 (PMC12712272; doi:10.1111/jmft.70104)
Supplement: Supplementary file 1 — Supplemental‐File‐Clean‐Unlinked. [file JMFT-52-0-s001.docx]

**How do couple relationship interventions improve individual well-being? The role of relationship confidence – Supplemental Material**

**Post-Hoc Analyses**

Two post hoc analyses were conducted related to changes in relationship confidence. These analyses were conducted using the packages lme4 (Bates et al., 2015) and lmerTest (Kuznetsova et al., 2017) in RStudio (R Core Team, 2023). To begin, we used a multilevel model with individuals (Level 1) nested within couples (Level 2) to examine individuals’ change in relationship confidence from pre- to post-program, accounting for the non-independence of data from partners within the same couple dyad. These results indicated a significant mean change in relationship confidence (*t*(461) = 7.61, *p* < .01), such that participants reported improved levels of relationship confidence at post-program compared to pre-program. This difference (i.e., improvement) possessed a medium effect size (*d* = .63).

As a second post-hoc analysis with relationship confidence, we used a multi-level linear regression model to examine sociodemographic predictors of improvements in relationship confidence; these analyses permitted the investigation of whether certain individuals or relationship types were more likely to experience changes in relationship confidence over the course of the program. Of the five sociodemographic factors tested in the model, results indicated marital status as the only significant predictor. Direction of coefficients indicated married individuals reported greater improvements in relationship confidence at the end of the program compared to nonmarried individuals (*B* = .77, *p* = .04). No significant effects were observed for sex, age, education level, or household income (see Supplemental Table 1 for details). In sum, these post-hoc analyses suggested that participants reported moderately strong increases in relationship confidence over the course of the program, with these improvements being similar for participants across a range of demographic factors (aside from marital status).

**Supplemental Material References**

Bates, D., Mächler, M., Bolker, B. M., & Walker, S. C. (2015). Fitting linear mixed-effects models using lme4. *Journal of Statistical Software*, *67*(1), 1–48. https://doi.org/10.18637/jss.v067.i01

Kuznetsova, A., Brockhoff, P. B., & Christensen, R. H. B. (2017). lmerTest package: Tests in linear mixed effects models. *Journal of Statistical Software*, *82*(13), 1–26. https://doi.org/10.18637/JSS.V082.I13

R Core Team. (2023). *R: A Language and Environment for Statistical Computing* (2023.06.02). R Foundation for Statistical Computing.

**Supplemental Table 1.** *Sociodemographic Predictors of Pre- to Post-Program Improvements in Relationship Confidence* (N = *269*)

|  | Relationship Confidence (W2) | |  |
| --- | --- | --- | --- |
|  | *B* | *SE* |  |
| Female^1^ | 0.27 | .27 |  |
| Age | -0.01 | .01 |  |
| Income | 0.02 | .06 |  |
| Education level | 0.06 | .09 |  |
| Married^2^ | 0.77* | .36 |  |
| Relationship confidence (W1) | 0.60** | .04 |  |

*Note.* ^1^ 1 = female / 0 = male. ^2^ 1 = married; 0 = dating or engaged. W1 = Wave 1; W2 = Wave 2. * *p* <.05; ** *p* <.01.
